# Naked-Eye 3D Display Based on Microlens Array Using Combined Micro-Nano Imprint and UV Offset Printing Methods

**DOI:** 10.3390/molecules25092012

**Published:** 2020-04-25

**Authors:** Linyi Chen, Guangxue Chen, Liyu Liao, Haozhi Chen

**Affiliations:** 1 State Key Laboratory of Pulp and Paper Engineering, South China University of Technology, Guangzhou 510640, China; chen_lin_lin_yi@163.com (L.C.); chengx@scut.edu.cn (G.C.); 2 YUTO R&D Institute, Shenzhen YUTO Packaging Technology Co., Ltd., Shenzhen 518108, China; liaoly@szyuto.com; 3 Guangzhou Financial Service Innovation and Risk Management Research Base, South China University of Technology, Guangzhou 510640, China

**Keywords:** microlens array, micro-nano imprint, UV offset printing, naked-eye 3D display

## Abstract

An optical film integrating microlens array (MLAs) and 3D micro-graphics is an important way to achieve the naked-eye 3D display effect. The 3D micro-graphics is traditionally generated by the micro-nano imprint technology based on precision engraving mold, which leads to high production cost and low production efficiency, and thus restricts the rapid response to production tasks and large-scale popularization and application. In this study, a process scheme for large-scale printing of 3D micro-graphics using UV offset printing based on presensitized (PS) plate was proposed, matching with the MLAs fabricated by micro-nano imprint process to achieve naked-eye 3D display effect. We used the laser confocal microscope to systematically measure and analyze the geometric and optical performance of the fabricated MLAs in terms of height, curvature radius, center distance, spacing, focal length, and numerical aperture, and evaluated the influence of the publishing resolution of the PS plate on the display effect of 3D micro-graphics. The printing quality and display effect of 3D micro-graphics were further improved by adjusting process parameters such as printing speed and printing pressure. The results of the current study demonstrate that the combined application of micro-nano imprint technology based on precision mold and UV offset printing technology based on PS plate can achieve an excellent naked-eye 3D display effect in 360° all angles, which is efficient, cost-saving, and highly flexible.

## 1. Introduction

Microlens array (MLAs), as an important micro-optics component, has been widely used in optical communications, optical coupling [1,2], optical fluids [3], micro-optical sensors [1], lighting [1,4], imaging, and display [1,5,6]. Therefore, many fabrication techniques of MLAs have been proposed to satisfy various application requirements of different scenarios, which can be placed into several categories: chemical synthesis method [7,8], optofluidic method [9,10], surface tension effect assisted technology [11,12,13,14,15,16,17,18,19,20,21], graphic transfer technology [22,23,24,25,26,27,28,29], mechanical manufacturing technology [30,31,32,33], and photolithography assisted technology [34,35,36,37,38,39,40].

The application of MLAs in the printing industry has pushed the way of graphic imaging and display gradually from 2D to 3D. 3D printing technology based on MLAs can achieve naked-eye 3D effect in 360° all angles without any special observation equipment or skills, which can significantly improve the information display level, interest, and anti-counterfeiting performance of printed products, increase the visual impact and sensory experience, and greatly enhance the added value of printed products.

As a typical micro-nano processing technology, micro-nano imprint is seen as a common method to replicate micro-nano structures and MLAs among considerable MLAs fabrication methods. Although the geometric dimension of MLAs can only be changed by transforming the imprint mold, it can be applied to achieve stable production of large-area and large-scale MLAs on flexible substrates, such as polyethylene terephthalate (PET) film [24,25,26,27,28]. After the fabrication of the optical film with MLAs is completed, the designed 3D micro-graphics needs to be transferred on the optical film substrate and then matched with the MLAs to achieve the naked-eye 3D display effect. At present, micro-nano imprint based on precision engraving mold is a popular method of 3D micro-graphics transfer in the worldwide, which is universally adopted by the internationally renowned 3D printing company, including American Crane, Germany’s G&D, and Sweden’s Rolling Optics. However, this micro-graphics transfer method leads to high production cost and low production efficiency and thus restricts the rapid response to production tasks and large-scale popularization and application.

UV offset printing based on presensitized (PS) plate, which is globally the dominant printing method, has abundant advantages such as fast printing speed, short printing cycle, low printing cost, relatively stable printing quality, fine graphics and rich levels, etc. In this paper, UV offset printing process based on PS plate was used for the first time to transfer and replicate designed 3D micro-graphics, matching with the MLAs fabricated by micro-nano imprint process, to achieve the naked-eye 3D display effect. The surface flatness and light transmittance of the PET film were observed. The geometric and optical performance of the fabricated MLAs were characterized. The influence of computer to plate (CTP) publishing resolution on the imaging quality and 3D display effect of 3D micro-graphics was analyzed. The optimized printing speed and printing pressure range were obtained for improving printing quality and display effect of 3D micro-graphics.

## 2. Results and Discussion

### 2.1. Surface Flatness and Light Transmittance of PET Film

**Figure 1:**
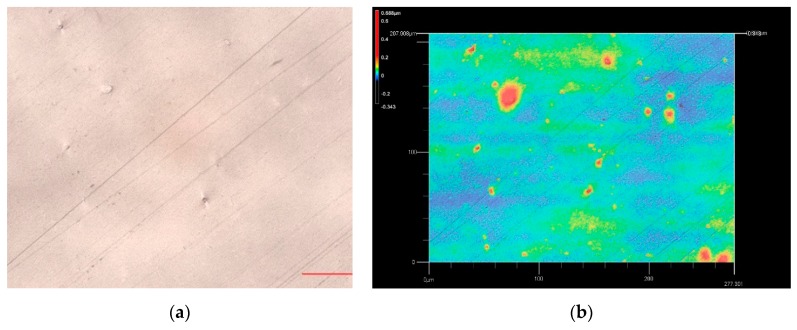

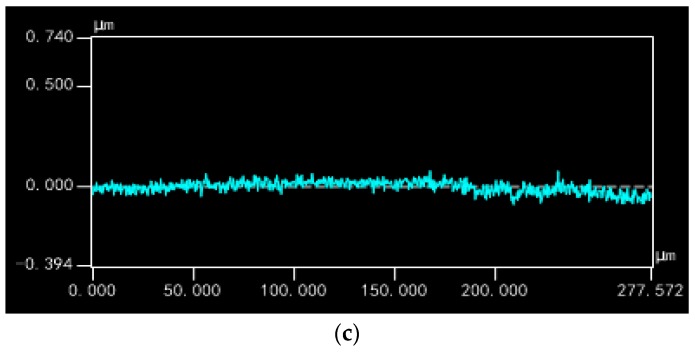
Geometric morphology and surface roughness of the polyethylene terephthalate (PET) film; (**a**) the surface morphology of the PET film; (**b**) the surface roughness of the PET film; (**c**) the cross-section of the PET film.

**Figure 2:**
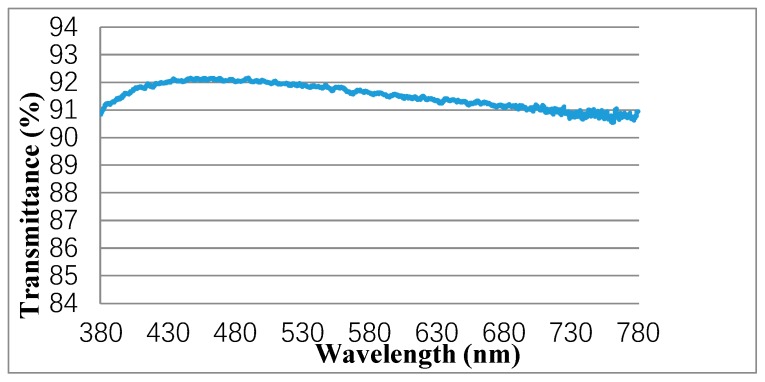
Visible light transmittance curve of the PET film.

As the fabrication substrate of the MLAs, surface flatness, and light transmittance of the PET film have significant impact on the preparation quality and optical characteristics of the MLAs. A laser confocal microscope was used to observe the surface and cross-section morphology of the PET film at a magnification of 3000×. The test results were shown in Figure 1. Meanwhile, the RMS (Root Mean Square) value of the surface roughness was calculated. As shown in Figure 2, the light transmittance of the PET film was measured using an ultraviolet spectrophotometer in the wavelength range of 380–780 nm, with a step size of 1 nm.

As seen in Figure 1a,b, the surface of the PET film was relatively clean and had almost no raised points. From the perspective of surface roughness, the calculated RMS value was 0.083 um (RMS values of surface roughness of other two brands of PET films were also measured and calculated. The measured results were about 0.2–0.3 um, which were all greater than what we used). As we can see from Figure 1c, the PET film had almost no jumping spikes, and the up and down fluctuations were relatively stable, which indicated that the PET film had good surface flatness. As shown in Figure 2, the light transmittance curve of the PET film fluctuated little and showed a high light transmittance. The average light transmittance was about 91–92%. The results in Figure 1 and Figure 2 demonstrated that the PET film had good surface flatness and light transmittance, which was quite beneficial to the fabrication of the MLAs and the presentation of the excellent optical performance of the 3D optical film.

### 2.2. Geometric and Optical Performance of MLAs

**Figure 3:**
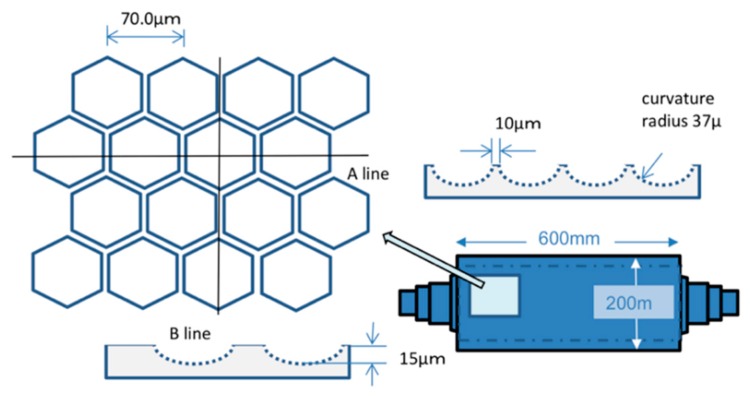
The schematic diagram of the microstructure of the precision optical mold.

The design parameters of the precision optical mold determine the shape, size, and arrangement of the microlens and thus determine the diameter, height, spacing, curvature radius, focal length, and numerical aperture of the microlens. Figure 3 shows the schematic diagram of the microstructure of the optical mold, which is a nickel alloy cylindrical seamless optical roller. The microlens unit is a regular hexagon, arranged in a regular hexagon. The center distance of the microlens is 70 um, the height of the microlens is 15 um, the spacing of the microlens is 10 um, and the curvature radius of the microlens is 37 um.

**Figure 4:**
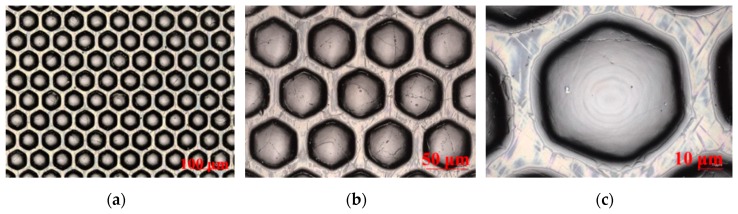

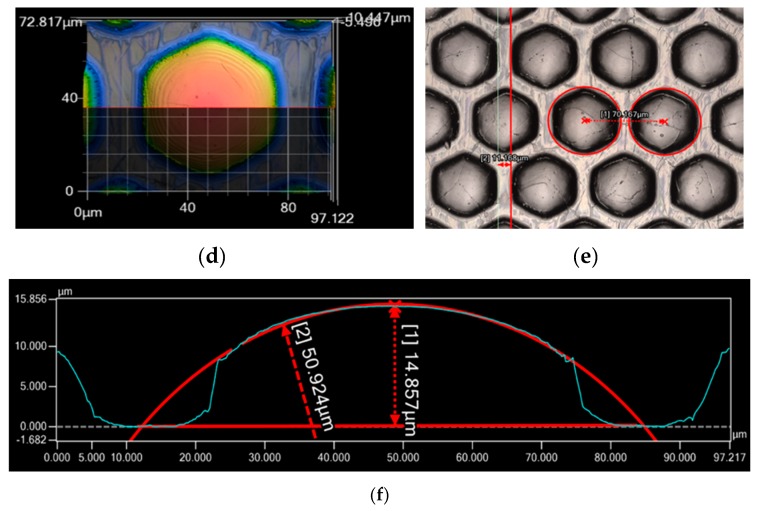
Plane morphology, 3D morphology, and geometric dimension of the MLAs; (**a**–**c**) the plane morphology of the MLAs at the magnifications of 400×, 1000×, and 3000×, respectively; (**d**) the 3D morphology of the MLAs at the magnification of 3000×; (**e**) the marked test picture of the center distance and spacing of the microlens; (**f**) the marked test picture of the height and curvature radius of the microlens.

When the optical mold, the optical film substrate, and the UV photoresist are fixed, the fabrication quality of the MLAs is mainly determined by the imprint speed and UV curing temperature. Extensive tests at different imprint speeds and UV curing temperatures have been conducted. We found that the fabrication quality was stable and better when the imprint speed was controlled at 5 m/min to 8 m/min and the curing temperature was controlled at 60–80 °C. In UV curing of MLA, the UV lamp power was 2500 mJ, and the exposure time was 2–4 s. Subsequently, 3D optical film fabricated at an imprint speed of 5 m/min, and curing temperature of 70 °C was taken as an example to characterize the geometric and optical performance of its MLAs. We randomly cut a 3 cm × 3 cm 3D optical film and used the laser confocal microscope to obtain its plane and 3D morphology and marked the test lines of the center distance, spacing, height and curvature radius of the microlens, as shown in Figure 4.

We can intuitively see from Figure 4a–d, the overall fabrication effect of the MLAs was good, but there were several fine cracks on the surface, which may be caused by the internal stress due to the instant separation of the photoresist after UV curing. According to Figure 4e–f, the average values of the center distance, spacing, height, and curvature radius of all microlenses in the cutting range were measured and calculated and then compared with the original design values. The calculation and comparison results were shown in Table 1.

As seen from Table 1, the absolute values of the deviation rate of the center distance, spacing, height, and curvature radius were 0.39%, 1.80%, 1.20%, and 10.14%, that is, the values of the center distance, spacing, and height were not significantly different from the original design values, except that the curvature radius was slightly larger, which indicated that the MLAs had good uniformity and high cycle accuracy.

Focal length and numerical aperture, as the most important evaluation parameters of the optical performance for the MLAs, can be described as the formula 1 and 2 [13,14].

(1) $$f=\frac{R_{c}}{n-1}$$

(2) $$NA=\frac{D}{2f}$$

where *Rc* is the curvature radius of the microlens, *n* is the reflection coefficient of the microlens, and the value is 1.50; *D* is the diameter of the microlens. According to the measured values of the curvature radius and center distance of the microlens in Table 1, combined with the above formula, the focal length and numerical aperture of the MLAs can be calculated as 81.50 um and 0.43, respectively, which demonstrated that the MLAs had a good optical performance.

### 2.3. The Influence of CTP Publishing Resolution on the Imaging Quality and 3D Display Effect of 3D Micro-Graphics

**Figure 5:**
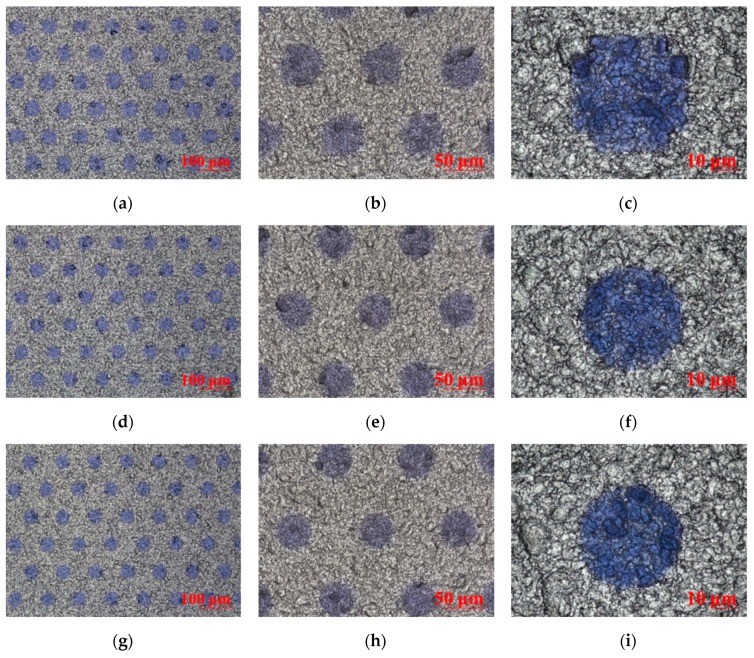
Geometric morphology of the presensitized (PS) plates with three resolutions at the magnifications of 400×, 1000×, and 3000×; (**a**–**c**) the morphology of the 3200 dpi PS plate at the magnifications of 400×, 1000×, and 3000×; (**d**–**f**) the morphology of the 6400 dpi PS plate at the magnifications of 400×, 1000×, and 3000×; (**g**–**i**) the morphology of the 12,800 dpi PS plate at the magnifications of 400×, 1000×, and 3000×.

**Figure 6:**
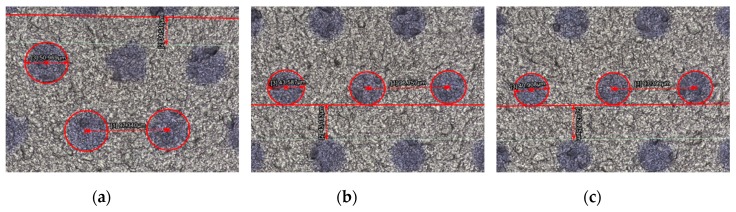
The marked test pictures of 3D micro-graphics dimensions of the PS plates with three different resolutions; (**a**–**c**) 3200 dpi, 6400 dpi, and 12,800 dpi successively.

**Figure 7:**
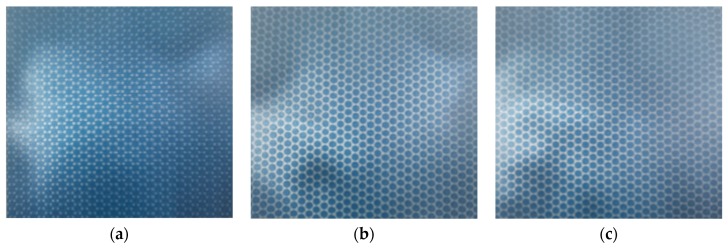
Comparison of 3D imaging effects of three different resolutions in the mode where the PS plate and the 3D optical film were overlaid; (**a**–**c**) 3200 dpi, 6400 dpi, and 12,800 dpi successively.

The quality of CTP publishing plays a crucial role in the imaging effect of 3D micro-graphics and the final naked-eye 3D display effect, and the publishing resolution is one of the decisive factors for CTP publishing quality. Kodak CTP platesetters were used to publish PS plates at 3200 dpi, 6400 dpi, and 12,800 dpi resolutions. The design requirement for publishing documents: the dot shape is circular, arranged in a regular hexagon, the diameter is 40.5 um, the center distance is 97 um, and the line spacing is 43.5 um. The geometric morphology and 3D graphics dimension of the PS plates with different resolutions were tested and analyzed using the laser confocal microscope. Figure 5 showed the plan morphology of the PS plate with three resolutions of 3200 dpi, 6400 dpi, and 12,800 dpi at magnifications of 400×, 1000×, and 3000×. Figure 6 showed 3D micro-graphic dimensions of the PS plate with three different resolutions. Table 2 showed the comparison results between the measured and the original design values of the 3D micro-graphics at three different resolutions. Finally, the imaging and display effects of 3D micro-graphics at different resolutions were observed in the mode where the PS plate and the 3D optical film were overlaid, as shown in Figure 7.

As seen from Figure 5, the smaller the publishing resolution, the easier the dot gain phenomenon of micro-graphics occurred. As shown in Figure 6 and Table 2, the measured values of micro-graphics dimensions (including the center distance, line spacing, and diameter) at various resolutions had different degrees of deviation from the original design values. We can calculate from Table 2 that the absolute values of the deviation rates of the center distance, line spacing diameter compared with the original design values at 3200 dpi were 0.35, 20.58, 25.85; 0.25, 0.73, 7.62 at 6400 dpi; and 0.41, 1.44, 1.01 at 12,800 dpi. The average values of the absolute values of the deviation rates of the center distance, line spacing, and diameter of the micro-graphics at 3200 dpi, 6400 dpi, and 12,800 dpi were thus calculated to be 15.59, 2.87, and 0.95, respectively, which indicated that the micro-graphics dimensions at 12,800 dpi were closest to the original values, and there was almost no deviation.

We can intuitively see from the comparison of the imaging effects in Figure 7 that the micro-graphics become more and more delicate, and the distortion of the micro-graphics was less obvious as the publishing resolution increased.

### 2.4. UV Offset Printing Process Control of 3D Micro-Graphics

The 3D optical film fabricated at an imprint speed of 5 m/min and curing temperature of 70 °C was used as a printing substrate, and the PS plate with a publishing resolution of 12,800 dpi was used as a printing plate. Abundant tests for 3D micro-graphics transfer were conducted by the UV label offset printing machine. We found that the printing quality of 3D micro-graphics and the naked-eye 3D display effect were relatively best when the printing speed was controlled at 20–30 m/h and the printing pressure was controlled at 0.05–0.15 Pa. Subsequently, a microlens 3D printed sample produced at a printing speed of 23 m/h and a printing pressure of 0.1 Pa was used as an example to observe and analyze the geometric morphology of the 3D micro-graphics by the laser confocal microscope.

**Figure 8:**
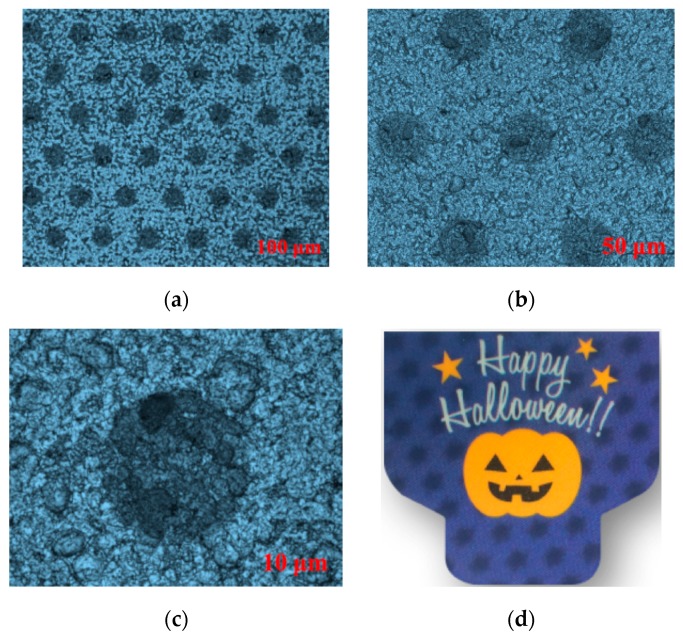
Microlens 3D printing sample and geometric morphology of 3D micro-graphics at the magnifications of 400×, 1000×, and 3000×; (**a**–**c**) the morphology of 3D micro-graphics at the magnifications of 400×, 1000×, and 3000×, respectively; (**d**) the effect picture of microlens 3D printing sample.

As seen from Figure 8a–c, although the round dot was slightly faint, there was almost no deformation and no obvious dot gain. As shown in Figure 8d, the sample showed a good naked-eye 3D display effect, which indicated that the offset printing based on PS plate can realize the high-quality transfer of 3D micro-graphics, and match with the MLAs to achieve an excellent naked-eye 3D display effect.

**Figure 9:**
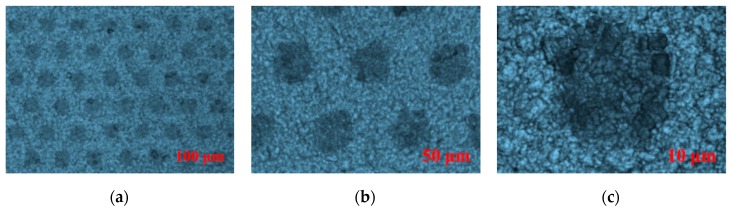

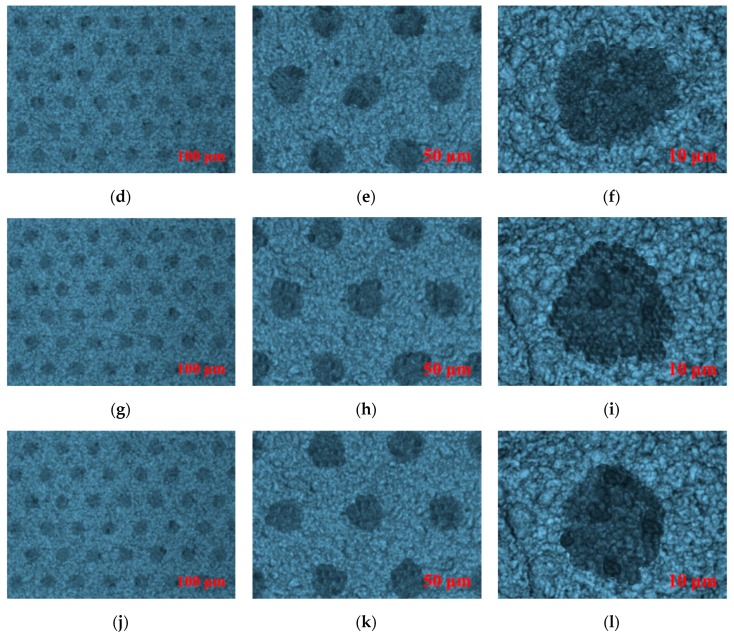
Geometric morphology of 3D micro-graphics for 4 groups of production condition at the magnifications of 400×, 1000×, and 3000×; (**a**–**c**) 15 m/h, 0.03 Pa; (**d**–**f**) 15 m/h, 0.1 Pa; (**g**–**i**) 35 m/h, 0.1 Pa; (**j**–**l**) 35 m/h, 0.2 Pa.

The microlens 3D printed samples produced at 4 groups of production conditions outside the optimal parameter range were used to observe and analyze the geometric morphology of the 3D micro-graphics by the laser confocal microscope, as shown in Figure 9. The 4 groups of production condition were, respectively, 15 m/h, 0.03 Pa; 15 m/h, 0.1 Pa; 35 m/h, 0.1 Pa; and 35 m/h, 0.2 Pa.

We can see from Figure 9 that the changes in printing speed and printing pressure had a significant impact on the quality of 3D micro-graphics printing and naked-eye 3D display. Whether the printing speed or printing pressure became larger or smaller, the phenomenon of dot gain or graphics blurring always existed. Consequently, like ordinary paper-based UV offset printing, it is required to flexibly adjust the corresponding printing speed and printing pressure according to the performance of microlens 3D optical film and other printing consumables in the process of UV offset printing based on microlens 3D optical film so as to achieve the best matching state between the 3D printing material, printing process, and printing equipment, in order to obtain unexpected 3D display effect.

### 2.5. Comparison of Micro-Nano Imprint and UV Offset Printing

The fabricated 3D optical film was used as a printing substrate. Micro-nano imprint and UV offset printing were applied to produce microlens 3D printed products, respectively. The production procedures of micro-nano imprint include 3D micro-graphics design, mold-making, imprint, and die cutting. Moreover, for UV offset printing, the production procedures include 3D micro-graphics design, PS plate-making, UV offset printing, and die cutting. Table 3 showed comparison between micro-nano imprint based on the precision engraving mold and UV offset printing based on PS plate in terms of efficiency, cost, and flexibility.

As seen from Table 3, compared with micro-nano imprint based on the precision engraving mold, the production efficiency based on UV offset printing has improved approximately 90%, and the production cost has reduced about 33%. Moreover, UV offset printing, in terms of flexibility, can realize personalized design of 3D micro-graphics according to customer needs, rapid plate-making, and fast response to production tasks.

## 3. Materials, Equipment, and Methods

### 3.1. Materials and Equipment

The experimental materials and their models, manufacturers, components, and properties are shown in Table 4.

The experimental equipment and their models, manufacturers, and functions are shown in Table 5.

### 3.2. Methods

#### 3.2.1. Fabrication of Microlens 3D Optical Film

Microlens 3D optical film was prepared by a micro-structured optical film fabrication machine using micro-nano imprint process. The operating environment of the machine usually needs to meet the following requirements: the cleanliness of the site must meet the standard of 10,000-level purification workshop; constant temperature and humidity environment, where the temperature is 23 °C ± 3 °C and the humidity is 45–65% RH (Relative Humidity); the environmental illumination is 200–250 LUX, and the light source is yellow during normal production; the workshop floor is anti-static.

**Figure 10:**
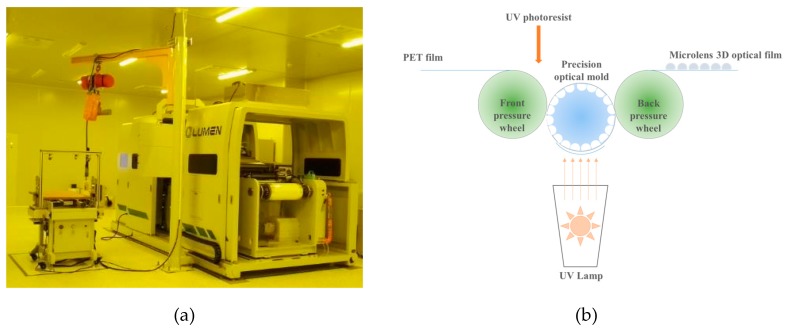
The fabrication machine and the schematic diagram of its film preparation principle; (**a**) the micro-structured optical film fabrication machine; (**b**) the preparation principle of optical film.

The preparation principle of the machine is as follows: the photoresist (ultraviolet-curable acrylic resin) flows into the micro-structured groove on the surface of the precision optical mold, the excess resin is squeezed out of the groove by the front pressure wheel, making the mold surface closely adhere to the PET film. The resin in the groove is cured on the surface of the PET film by a UV lamp. The film is peeled off from the mold after passing the backpressure wheel. The MLAs layer is firmly attached to the surface of the PET film finally to form a microlens 3D optical film. The micro-structured optical film fabrication machine and the schematic diagram of its preparation principle are shown in Figure 10.

**Figure 11:**
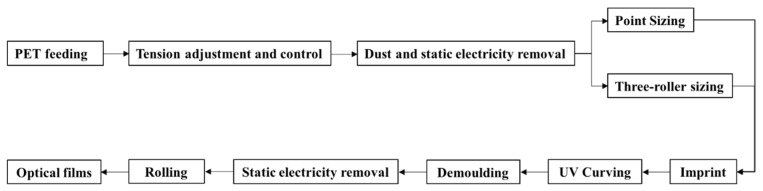
The detailed preparation process flow of the fabrication machine.

The detailed preparation process flow of the fabrication machine is shown in Figure 11.

It is required to remove the oil and impurities on the surface of the PET substrate with acetone, alcohol, or deionized water before sizing, and do antistatic treatment to ensure that the resin will be formed on the substrate surface uniformly and firmly. Furthermore, the speed of sizing must be strictly controlled to ensure the uniformity and flatness of the photoresist, avoiding the phenomenon of air bubbles caused by too fast sizing, or the phenomenon of overflow caused by too slow sizing.

#### 3.2.2. Design of 3D Micro-Graphics and Preparation of PS Plate

The imaging effect of 3D micro-graphics is a periodic superimposed 3D depth-of-field pattern generated by the superimposition of a new periodic moiré pattern and a microlens visual depth-of-field effect generated by the superposition of two arrays with similar periods. We assume that the distance between two adjacent microlenses of the 3D optical film is *T1*, the distance between the two adjacent elements of the designed 3D micro-graphics is *T2*, and the distance of the finally generated 3D imaging pattern is *T*, then the relation between the three variables can be described as the formula 3.

(3) $$=\frac{T_{1}\times T_{2}}{\left|T_{1}-T_{2}\right|}$$

Once the 3D optical film is fabricated, T1 can be determined. Adobe Illustrator was used to simulate the 3D imaging effect, and the 3D micro-graphics publishing file was designed after the imaging requirement matching the MLAs was met. A PS plate with 3D micro-graphics was then prepared by CTP platesetters.

#### 3.2.3. 3D Micro-Graphics Printing Based on PS Plate

**Figure 12:**
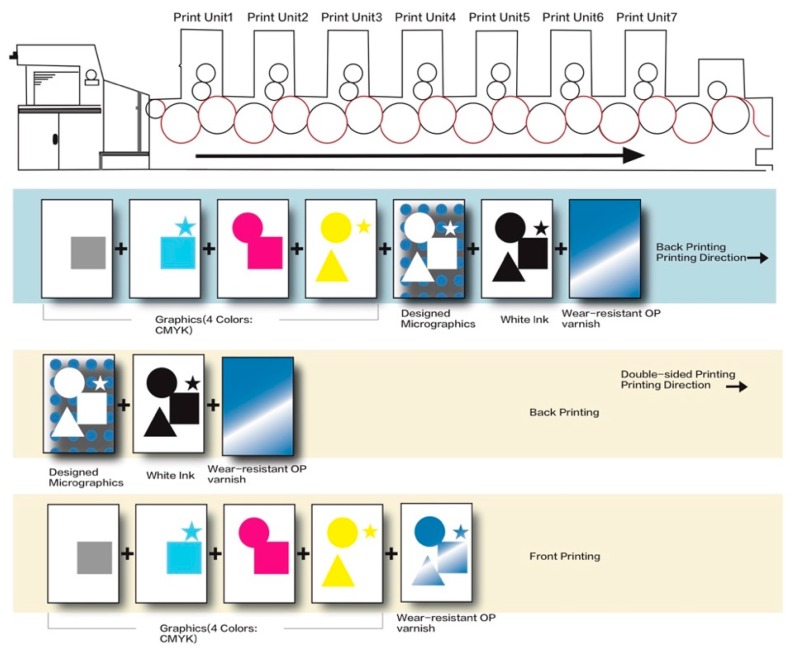
The schematic diagram of UV offset printing process of 3D micro-graphics.

The 3D label offset printing machine was used to transfer and replicate the 3D micro-graphics, with PS plate as printing plate and microlens 3D optical film as printing substrate. Generally, two printing methods can be used: one is printing on the back of the 3D optical film, and the other is printing on both sides of the 3D optical film. The schematic diagram of UV offset printing process of 3D micro-graphics was shown in Figure 12. For back printing, the printing color sequence is: four-color (C, M, Y, K) printing for surface graphics, 3D micro-graphics, white ink, wear-resistant overprint (OP) varnish. For double-sided printing, the printing color sequence is: 3D micro-graphics, white ink and wear-resistant OP varnish on the back of the 3D optical film firstly, and then four-color (C, M, Y, K) printing for surface graphics and wear-resistant OP varnish on the front of the 3D optical film.

**Figure 13:**
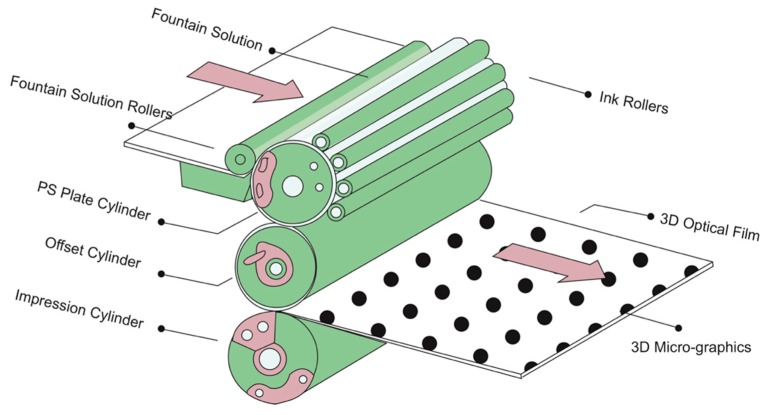
The detailed procedure diagram of 3D micro-graphics transfer.

The detailed procedure about how the 3D micro-graphics is transferred to the transparent 3D optical film was shown in Figure 13. The fountain solution is sized on the blank area of the PS plate through the fountain solution rollers, and the ink is then sized on the 3D micro-graphics area of the PS plate through the ink rollers. The 3D micro-graphics is firstly transferred to the rubber blanket through the rolling between the PS plate cylinder and the offset cylinder. Subsequently, the 3D micro-graphics is transferred to the 3D optical film by the rolling between the offset cylinder and the impression cylinder. In the process of transfer, the dot gain value should not be greater than 12%, and the overprint error should be less than 0.03 mm.

Compared with the existing micro-nano imprint based on the precision engraving mold, UV offset printing based on PS plate is highly efficient, cost-saving, and highly flexible and can respond quickly to production tasks, because it can take advantage of the high-precision and high-efficiency publishing capabilities of the CTP platesetters to quickly prepare the PS plate with 3D micro-graphics, and then easily and completely replicates the personalized micro-structured graphics to the transparent 3D optical film.

#### 3.2.4. Characterization Methods

The surface flatness of the PET film was observed by a laser confocal microscope. The light transmittance of the PET film was measured using an ultraviolet spectrophotometer in the wavelength range of 380–780 nm with a step size of 1 nm. The geometric and optical performance of the fabricated MLAs were characterized by a laser confocal microscope. The geometric morphology and 3D micro-graphics dimension of the PS plates with different publishing resolutions were tested by a laser confocal microscope, and the influence of CTP publishing resolution on the imaging quality and 3D display effect of 3D micro-graphics was analyzed. The optimized printing speed and printing pressure range were obtained for improving printing quality and display effect of 3D micro-graphics. The geometric morphology of the microlens 3D printed sample was observed with a laser confocal microscope.

## 4. Conclusions

UV offset printing process based on PS plate was used to transfer and replicate designed 3D micro-graphics, matching with the MLAs fabricated by micro-nano imprint process, to achieve the naked-eye 3D display effect. Compared with the existing micro-nano imprinting method based on the precision engraving mold for 3D micro-graphics transfer, UV offset printing based on PS plate is efficient, cost-saving, and highly flexible and can respond quickly to production tasks. The PET film had good surface flatness and light transmittance, which was quite beneficial to the fabrication of the MLAs and the presentation of the excellent optical performance of the 3D optical film. The fabrication quality of the MLAs was stable and better when the imprint speed was controlled at 5–8 m/min and the curing temperature was controlled at 60–80 °C. The absolute values of the deviation rate of the center distance, spacing, height, and curvature radius of the MLAs fabricated at the imprint speed of 5 m/min and the curing temperature of 60 °C were 0.39%, 1.80%, 1.20%, and 10.14%. The center distance, spacing, and height were not significantly different from the original design, except that the curvature radius was slightly larger, which indicated that the MLAs had good uniformity and high cycle accuracy. The average values of focal length and numerical aperture of the fabricated MLAs were 81.50 um and 0.43, respectively, which indicated that the MLAs had a good optical performance. In the mode where the PS plate and the 3D optical film were overlaid, the micro-graphics become more and more delicate, and the distortion phenomenon of the micro-graphics, such as dot gain, was less obvious as the publishing resolution increased. The publishing resolution of 3D micro-graphics generally should be chosen at above 12,800 dpi. The printing quality of 3D micro-graphics and the naked-eye 3D display effect were relatively best when the printing speed was controlled at 20–30 m/h and the printing pressure was controlled at 0.05–0.15 Pa. The microlens 3D printed sample produced at a printing speed of 23 m/h, and a printing pressure of 0.1 Pa showed good geometric morphology and 3D display effect. Compared with micro-nano imprint based on the precision engraving mold, the production efficiency based on UV offset printing has improved approximately 90%, and the production cost has reduced about 33%. Moreover, UV offset printing, in terms of flexibility, can realize personalized design of 3D micro-graphics according to customer needs, rapid plate-making, and fast response to production tasks. In conclusion, the combined application of micro-nano imprint technology based on precision mold and UV offset printing technology based on PS plate can achieve an excellent naked-eye 3D display effect in 360° all angles, which is efficient, cost-saving, and highly flexible.

## Figures and Tables

**Table 1:** Measured values of the geometry dimensions of the microlens and comparison results with original design values.

Parameter Name	Measured Value/um	Original Design Value/um	Absolute Value of Deviation Rate
Center distance of the microlens	70.27	70	0.39%
Spacing of the microlens	10.18	10	1.80%
Height of the microlens	14.82	15	1.20%
Curvature radius of the microlens	40.75	37	10.14%

**Table 2:** Comparison between the measured values and the original design values of 3D micro-graphics dimensions of the PS plates with three different resolutions.

Parameter Name	3200 dpi PS Plate/um	6400 dpi PS Plate/um	12,800 dpi PS Plate/um	Original Design Values/um
Center distance	97.34	96.76	97.40	97
Line spacing	34.55	43.18	42.88	43.5
Diameter	50.97	43.59	40.91	40.5

**Table 3:** Comparison between micro-nano imprint based on the precision engraving mold and UV offset printing based on PS plate.

Technology Name	Efficiency	Cost	Flexibility
Micro-nano imprint based on the precision engraving mold	approximately 22 days of production cycle (1 day for micro-nano graphics design, 20 days for mold-making, 1 day for imprint and die cutting)	about 30 RMB/m^2^	Personalized customization of 3D micro-graphics and mold-making cannot quickly realized according to customer needs or production tasks, difficulty in mold-making (whether cycle or cost).
UV offset printing based on PS plate	approximately 3 days of production cycle (1 day for micro-nano graphics design, 1 day for PS plate-making, 1 day for printing and die cutting)	about 20 RMB/m^2^	Personalized design of 3D micro-graphics and rapid plate-making can be realized according to customer needs, fast response to production tasks

**Table 4:** Experimental materials and their introduction.

Name	Model	Manufacturer	Component	Property
Photoresist	RES-R136 −008	Poly-tech Material (Taiwan, China)	40–50% Acrylate resin monomer, 40–43% Acrylate resin oligomer, 3–5% 2-hydroxy-2-methylpropiophenone, 1–2% 1-hydroxycyclohexyl phenyl ketone, 0.01–1% Additional	viscosity is 250 cps @ 25 °C, reflectance is 1.5
PET film	Nan-ya	Nan-ya Plastics (Taiwan, China)	Polyethylene terephthalate	Light transmittance is above 90%, thickness is 75 um
PS plate	LH-PA ST	Fuji Japan	Thermal sun pattern CTP plate	Photosensitive wavelength is 830 nm, exposure energy is 120–140 mj/cm2, resolution is 200 lpi
UV offset printing ink	Bo-man HLED series	Dong-ya Chemical (Hongkong, China)	—	—

**Table 5:** Experimental equipment and their introduction.

Name	Model	Manufacturer	Function	Note
Micro-structured optical film fabrication machine	ALM-FD-I×00	A-Lumen (Taiwan, China)	For microlens 3D optical film fabrication	Fabrication of 3D optical films with a thickness of 38 um–188 um
Precision optical mold	UVM-363-1	A-Lumen (Taiwan, China)	As fabrication mold of MLAs	Cylindrical seamless optical roller made of nickel alloy prepared by laser engraving
CTP platesetters	Q400 UHR	Kodak Japan	For preparation of high-resolution PS plate	With Kodak T-MDE plate processor
Label offset printing machine	ZTJ330	Zhong-tian Printing (Ruian, China)	For 3D micro-graphics and color graphics printing	Multifunctional intermittent PS plate label printing machine
Laser scanning confocal microscope	VK-×1000	Keyence Japan	For observing the geometric characteristics and 3D dimension of the sample	—
UV spectrophotometer	UV-2600	Shimadzu Japan	For measuring the full-band transmittance and color coordinates of the sample	—
